# HMG-boxes, ribosomopathies and neurodegenerative disease

**DOI:** 10.3389/fgene.2023.1225832

**Published:** 2023-08-03

**Authors:** Tom Moss, Mark S. LeDoux, Colyn Crane-Robinson

**Affiliations:** ^1^ Laboratory of Growth and Development, St-Patrick Research Group in Basic Oncology, Cancer Division of the Quebec University Hospital Research Centre, Québec, QC, Canada; ^2^ Department of Molecular Biology, Medical Biochemistry and Pathology, Faculty of Medicine, Laval University, Québec, QC, Canada; ^3^ Department of Psychology, University of Memphis, Memphis, TN, United States; ^4^ Veracity Neuroscience LLC, Memphis, TN, United States; ^5^ Biophysics Laboratories, School of Biology, University of Portsmouth, Portsmouth, United Kingdom

**Keywords:** neuroregression syndrome, ribosomopathy, upstream binding factor (UBF/UBTF), ribosome biogenesis, RNA polymerase I (POLR1), TBP-TAF complex SL1, UBTF-E210K

## Abstract

The UBTF E210K neuroregression syndrome is a predominantly neurological disorder caused by recurrent *de novo* dominant variants in Upstream Binding Factor, that is, essential for transcription of the ribosomal RNA genes. This unusual form of ribosomopathy is characterized by a slow decline in cognition, behavior, and sensorimotor functioning during the critical period of development. UBTF (or UBF) is a multi-HMGB-box protein that acts both as an epigenetic factor to establish “open” chromatin on the ribosomal genes and as a basal transcription factor in their RNA Polymerase I transcription. Here we review the possible mechanistic connections between the UBTF variants, ribosomal RNA gene transcription and the neuroregression syndrome, and suggest that DNA topology may play an important role.

## Introduction

Several human medical disorders have been linked to variants in genes implicated in ribosome biogenesis, the synthesis and assembly of ribosomes. These disorders have therefore come to be known collectively as ribosomopathies. They range widely in their clinical presentations from disorders of the blood, skeleton, and neurological system to a variety of cancers. This pleiotropy appears at first sight to be at odds with the unique and discrete function of the ribosome in cellular protein synthesis. This said, ribosome biogenesis is a highly complex process involving the assembly of more than 80 ribosomal proteins onto a large structural and catalytic ribosomal RNA (rRNA) scaffold and requires the intervention of many hundreds of accessory proteins, hundreds of small RNAs, and extensive rRNA and rprotein modifications (Klinge and Woolford, 2019). Therefore, ribosome biogenesis can be affected in many ways and to differing degrees by variants in any of hundreds of genes. This in turn can lead to the activation or inhibition of key pathways that control growth, proliferation and senescence such as p53, RB, and Myc (Lessard et al., 2019). Since the ribosome is ultimately responsible for the translation of the cell’s genetic program, dysfunctions in ribosome biogenesis can not only affect cell and tissue growth but also metabolism, differentiation and development. Thus, despite the fundamental role of ribosomes in protein synthesis, ribosomopathies can and do present a surprisingly diverse range of phenotypes and tissue-specific effects. These span from Diamond-Blackfan Anemia (DBA), related Myelodysplastic Syndromes (MDS), Alopecia, Neurological Defects and Endocrinopathy Syndrome (ANES) to the cranial malformations of Treacher Collins Syndromes (TCSs) (Narla and Ebert, 2010; Aspesi and Ellis, 2019; Farley-Barnes et al., 2019; Orgebin et al., 2020), North American Indian childhood cirrhosis (NAIC) (Chagnon et al., 2002; Freed et al., 2012), and most recently to the UBTF E210K Neuroregression Syndrome also known as Childhood-Onset Neurodegeneration with Brain Atrophy (CONDBA) (Edvardson et al., 2017; Toro et al., 2018; Sedlackova et al., 2019; Bastos et al., 2020; Tinker et al., 2022). This phenotypic diversity and the complexity of ribosome biogenesis together often make it difficult to identify and understand the underlying cause of each disease. However, a small subset of ribosomopathies, despite having very different clinical outcomes, all point to dysfunctions at the level of rRNA gene transcription as the common factor.

## Ribosomopathies and rRNA gene transcription

**FIGURE 1:**
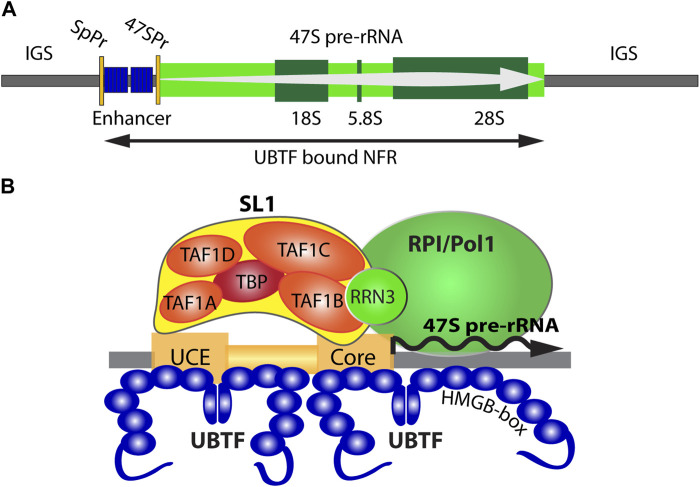
**(A)** Organisation of the transcriptionally active ribosomal RNA gene (rDNA) showing the 47S primary transcript, 18S, 5.8S and 28S coding regions, and the 47S promoter (47SPr) flanked upstream by the Enhancer region and its associated Spacer Promoter (SpPr). The nucleosomal Inter-Genic Spacer (IGS) is also shown flanking the UBTF-bound nucleosome-free region (NFR). **(B)** A schematic representation of the RNA polymerase I (RPI) preinitiation complex and the 47S promoter Upstream and Core regions.

Ribosome biogenesis starts in the nucleolus with the transcription of the rRNA genes (aka the rDNA), and ribosome assembly begins co-transcriptionally on these genes (Figure 1A). This makes the initiation of rDNA transcription the very first step in the process of ribosome biogenesis and hence the primary determinant of ribosome production (Figure 1B). Indeed, inhibition of transcription initiation on the rDNA prevents the assembly of new ribosomes, e.g., see (Claypool et al., 2004; Yuan et al., 2005; Herdman et al., 2017). Rare recessive variants in the POLR1A and POLR1B genes that encode the two largest subunits of RNA Polymerase I (RPI, PolI, POLR1), the polymerase uniquely responsible for rDNA transcription, were shown to cause a subset of TCS disorders (Weaver et al., 2015; Sanchez et al., 2020). Variants in TCOF1, a protein implicated in rDNA transcription in collaboration with the RPI basal factor Upstream Binding Factor (UBTF or UBF), are another cause of TCS (Lin and Yeh, 2009; Sakai and Trainor, 2009). A very rare recessive compound missense variant in TAF1A, a subunit of the RPI basal factor Selectivity Factor 1 (SL1), was also shown to cause cardiomyopathy [Long et al., 2017, NM_005681.4 (TAF1A):c.781A>C (p.Thr261Pro)]. Finally, variants in UBTF have been linked to degenerative neurological disease as well as to MDS (Edvardson et al., 2017; Toro et al., 2018; Sedlackova et al., 2019; Bastos et al., 2020; Umeda et al., 2022). Since the RPI machinery is used solely to synthesize the non-coding rRNAs, it is extremely probable that these variants all exert their primary effects at the level of rDNA transcription. In this context, UBTF is particularly interesting because of its bifunctionality as RPI basal transcription and as an epigenetic remodelling factor.

## UBTF genotype-phenotype associations

The UBTF E210K neuroregression syndrome, a distinct, predominantly neurological disorder, is caused by recurrent *de novo* dominant variants in UBTF (NM_014233.3:c.628G>A, p. Glu210Lys) (ClinVar SCV000598648.1) (Edvardson et al., 2017; Toro et al., 2018). Some affected individuals show slight developmental delay. Most affected individuals did tend to lose weight after 2 years. Neuroregression typically becomes apparent by 2.5–3 years of age and is global with decline in cognition, behavior, and sensorimotor functioning. Patients tend to lose previously acquired milestones after 3 years of age. Early motor dysfunction includes hypotonia, gait ataxia, and dysarthria. Later motor dysfunction is dominated by hypertonia with spasticity and dystonia, and postural instability with loss of ambulatory abilities. Most patients are non-ambulatory by their early teens. Individuals surviving to 30 years or more become bedbound, mute, and require gastrostomy tube placement to meet nutritional needs. Cognitive decline is first manifest as expressive dysphasia progressing to global aphasia. Some affected individuals also exhibit mild dysmorphic features and/or extra-neural manifestations. The phenotypic spectrum of patients with the E210K variant may include epilepsy (Sedlackova et al., 2019), and dystonia-Parkinsonism (Ikeda et al., 2021). Magnetic resonance imaging (MRI) shows progressive brain atrophy (supratentorial > infratentorial with gray matter > white matter). Gyral patterns are normal and there are no heterotopias, abnormal sulcation, or dysplastic cortical regions. Ex vacuo ventriculomegaly is apparent in older subjects. MRI findings are not characteristic of a leukodystrophy and suggest that demyelination is secondary.

Initial studies using patient fibroblasts suggested that variant E210K UBTF functions as a “hyperactive transcription factor” resulting in increased accumulation of 18S rRNA (Edvardson et al., 2017). ChIP-qPCR experiments showed nearly 3X increased presence of E210K UBTF across the regions of the rDNA promoter and the external transcribed spacer (ETS). This was associated with 4-fold higher accumulation of 18S rRNA. In follow-up work also using patient fibroblasts, the E210K UBTF variant was associated with increases in the accumulation of pre-rRNA (>3X) and 18S rRNA (>2X) compatible with a molecular gain-of-function mechanism (Toro et al., 2018). In contrast, in fibroblasts from a homozygous mouse knock-in E210K mutant model, ChIP-Seq revealed reduced association UBTF1 and SL1 precisely at the rDNA promoter and this correlated with a reduced rate of pre-rRNA synthesis as determined by metabolic labelling (Tremblay et al., 2022). In this context, it should be noted that rRNA accumulation is determined by a balance between the rates of synthesis, processing and degradation, all of which may be affected directly or indirectly by UBTF variants, whereas the metabolic labelling protocol determines the *de novo* pre-rRNA synthesis rate (Stefanovsky and Moss, 2016).

**FIGURE 2:**
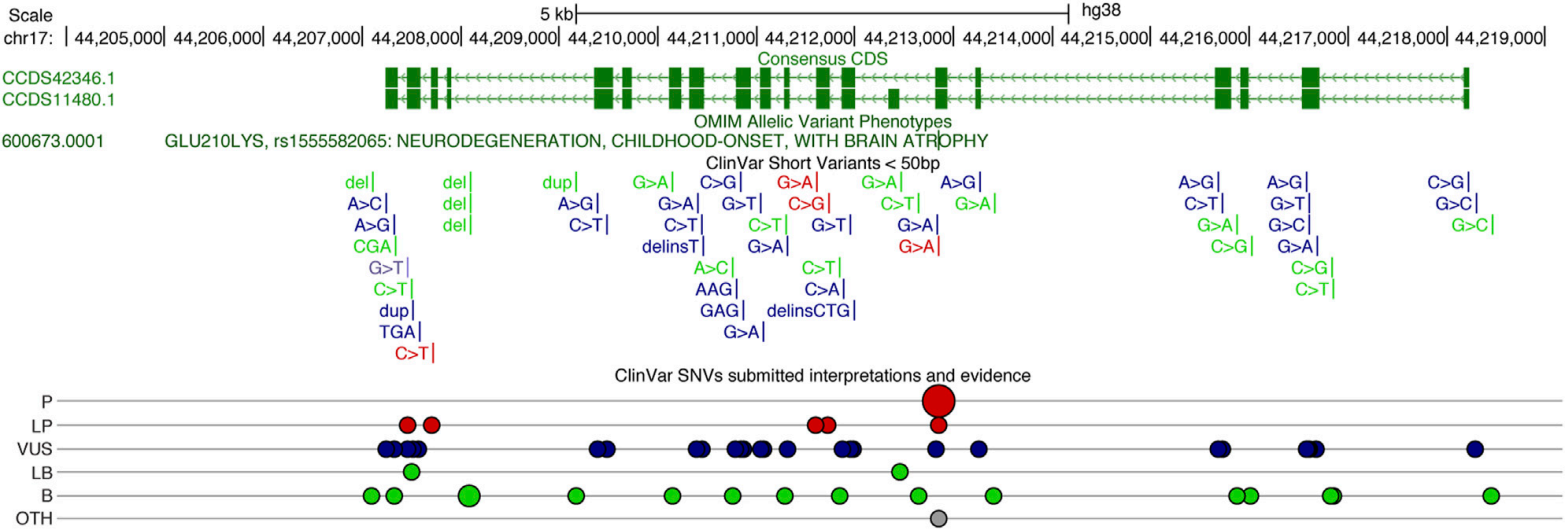
UCSC Genome Browser display of the Human *UBTF* locus on chr17 (http://genome.ucsc.edu), showing the consensus coding sequence (CCDS) annotations in green above the ClinVar variants (Landrum et al., 2018). Gains are noted in blue and losses in red. ClinVar single nucleotide variants (SNVs) classified (ClinVar interp) as P (Pathogenic), LP (Likely Pathogenic), VUS (Variant of Unknown Significant), LB (Likely Benign), B (Benign), and Other.

Other deleterious *UBTF* variants have been described in patients with neurological disease (Tinker et al., 2022) and reported in clinical-genetic databases (ClinGen and ClinVar). The p. Q203R variant (ClinVar SCV002001591.2) reported by Tinker and colleagues was associated with developmental delay, noted at 9 months of age, and striking neuroimaging abnormalities including pontine and thalamic hypoplasia. Other clinical findings included microcephaly (<1%) and hypotonia. To date, 25 missense variants have been submitted to ClinVar (Figure 2). Pathogenic, likely pathogenic, and variants of unknown significance cover most exons of *UBTF*, and most lie within UBTF structural domains of known significance. Large structural variants that encompass *UBTF*, including 3 duplications and 2 deletions are classified as pathogenic (Figure 2). In the gnomAD v2.1.1 dataset, there are no observed putative loss-of-function (pLoF) variants in *UBTF*. Based on ClinVar’s 4 April 2023 release, there are 42 UBTF variants. Of these, only 18 are also present in gnomAD v.3.1.2 and only 15 in gnomAD v2.1.1. Based on MetaLR and REVEL scores, many of the variants of unknown significance are likely to be highly deleterious to UBTF function.

Tandem duplications (TD) of *UBTF* (*UBTF*-TD) have also been identified in pediatric acute myeloid leukemia (AML) (Umeda et al., 2022). These variants are either in-frame insertions at the 3′end of exon 13 of *UBTF* or in-frame duplication of exon 13, and collectively referred to as UBTF-TD. They are extremely variable in length, affect HMGB-box 4 of UBTF (see below) for which little functional data is available, and can enhance growth when exogenously expressed in cord blood cells. *UBTF*-TDs are also seen in adult AML (Duployez et al., 2023). In both children and adults, patients harboring *UBTF*-TD tend to have poor outcomes (Kaburagi et al., 2023).

In mice, UBTF is essential for embryogenesis and survival in adults (Hamdane et al., 2014; Hori et al., 2022). Motor and cognitive assessment of *Ubtf* ^+/-^ mice showed that the deleterious effects of UBTF haploinsufficiency progress with age. No overt extra neural manifestations of UBTF deficiency were seen in mice. Work with mouse models and clinical-genetic data from humans indicates that both loss- and gain-of-function variants likely contribute to human disease. Moreover, at the most basal level, gain-of-function is invariably associated with some loss of normal function. UBTF has two major isoforms (UBTF1, UBTF2) that play distinct roles, see below, and certain genetic variants may exert differential effects on UBTF1 and UBTF2. Finally, the effects of UBTF gain or loss likely differ in post-mitotic neurons *versus* proliferating tissues (i.e., brain vs. hematopoietic system).

## The ribosomal RNA genes and their transcription

The human genome, like that of most eukaryotes, contains several hundred rDNA copies arranged in megabase tandem arrays. In human, these rDNA arrays are present on the short arms of the five acrocentric chromosomes and, since their transcriptional activity initiates the formation of the nucleoli, each array constitutes a Nucleolar Organizer Region (NOR). In human and mouse, the rDNA repeat units are 43 or 45 kbp in length and within each species are essentially identical (Grozdanov et al., 2003; Hori et al., 2021). Each rDNA repeat encodes the 18S, 5.8S, and 28S ribosomal RNAs in a single 47S rRNA precursor coding region of 13.3 or 13.4 kb whose transcription by RPI initiates the process of ribosome assembly (Figure 1A). RPI recruitment to the rDNA requires the formation of a Pre-Initiation Complex (PIC) consisting of the Selectivity Factor (SL1), consisting of the TATA-box Binding Protein (TBP) and the four TBP Associated Factors TAFIA to D, and the multi-HMGB-box Upstream Binding Factor (UBTF or UBF) (Moss et al., 2007) (Figure 1B). A third basal factor, RRN3, associates with RPI itself, enabling it to interact with the SL1/UBTF PIC and to initiate 47S synthesis, but is released soon after and recycled. SL1, RRN3 and UBTF are each essential for rRNA gene activity *in vivo* and are absolutely required for cell and organism viability (Hamdane et al., 2014; Herdman et al., 2017; Tremblay et al., 2022). Transcription by RPI can be highly organism- and species-specific, the coevolution of rDNA promoter sequences and the TAFI components of SL1 being the major, though perhaps not the sole, origin of this specificity. Thus, the human and mouse RPI promoters and their cognate SL1 complexes are not functionally interchangeable (Rudloff et al., 1994; Heix et al., 1997; Murano et al., 2014). In contrast, UBTF is a highly conserved factor, that is, functionally interchangeable between human, mouse and even to some extent amphibia (Hannan et al., 1999). This interchangeability is possibly a reflection of its very poor DNA sequence selectivity since it binds similarly to the rDNA promoters of different species and apparently unrelated primary sequence (Bell et al., 1989; Pikaard et al., 1990). Despite this, recent data has shown that UBTF plays at least two key roles in determining rDNA activity, 1) by determining a specialized non-nucleosomal chromatin structure on the active rDNA and 2) by cooperating in a structurally precise manner in PIC formation at the RPI promoter (Hamdane et al., 2015; Herdman et al., 2017; Moss et al., 2019; Tremblay et al., 2022).

## UBTF determines active rDNA chromatin

UBTF was the first RPI basal factor to be identified (Bell et al., 1990; Jantzen et al., 1990). However, it was later found to bind widely across the rDNA repeat (O'Sullivan et al., 2002) and more recently to delineate a Nucleosome-Free Region (NFR) across the 47S coding region and the 5′-proximal promoter and “enhancer” elements (Herdman et al., 2017; Moss et al., 2019; Tremblay et al., 2022) (Figure 1A). Thus, one function of UBTF was found to be the epigenetic remodel of rDNA chromatin, replacing canonical nucleosomes with an alternative UBTF-based structure (Moss et al., 2019; Tremblay et al., 2022). Many years ago, a series of Electron Spectroscopic Imaging (ESI) studies suggested that this UBTF nucleoprotein structure was similar in mass and DNA-protein composition to the nucleosome but was quite distinct in structure (Bazett-Jones et al., 1994; Stefanovsky et al., 1996; Stefanovsky et al., 2001).

**FIGURE 3:**
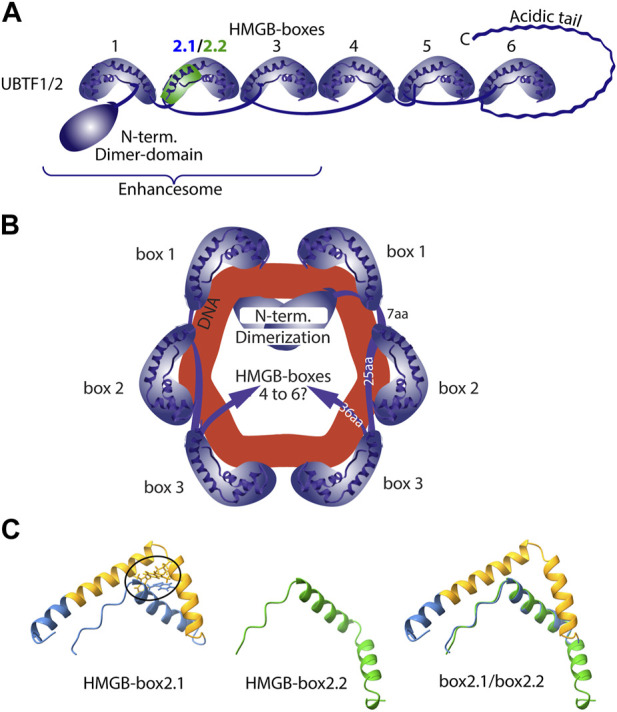
Structural models of the UBTF-DNA nucleoprotein complex. **(A)** The structural domains of UBTF1 and 2 variants. **(B)** The Enhancesome structure deduced from ESI studies on UBTF trunaction variants and indicating the bending of DNA, shown in red. **(C)** The AlphaFold predicted structure of HMGB-box 2.1. The central spliced subdomain is indicated in yellow, the hydrophobic core is ringed and the structure predication for the spliced box 2.2 is shown in green.

The UBTF protein consists of an N-terminal dimerization domain followed by the tandem organization of 6 HMGB-box homology domains (HMGB-boxes) and a highly acidic and probably unstructured C-terminal domain (Acidic Tail) (Figure 3A). HMGB-boxes are small sequence non-specific DNA binding domains most closely related in structure to the DNA interaction domains of HMGB1 and having the general property of bending DNA. ESI showed that the three N-terminal HMGB-boxes of UBTF induced adjacent in-phase DNA bends, such that a dimer of UBTF induced a single 360 deg. looping in about 140 bp of DNA (Figure 3B). This structure, called an *Enhancesome* due to its discovery on the rDNA enhancer repeats, was clearly incompatible with the canonical nucleosome. Hence, UBTF defined a novel form of chromatin that was responsible for the epigenetic reprogramming of the active rDNA. However, the involvement of this novel chromatin in PIC formation at the rDNA promoters remained until recently open to conjecture.

## Only the UBTF1 variant cooperates with SL1 in PIC formation

Human cell-free transcription assays had originally suggested a sequential model for RPI PIC assembly in which UBTF recruitment to the rDNA promoter provided a platform to which SL1 could bind. However, the inverse order of binding was also found to be possible (Friedrich et al., 2005), while in mouse and rat cell-free assays UBTF was found not to be essential (Kuhn and Grummt, 1992; Smith et al., 1993). In short, these early findings suggested that SL1 alone could drive PIC formation and left open the question of UBTF. This remained the case until *in vivo* studies reposition one of the two UBTF splice variants as the key factor in PIC formation (Tremblay et al., 2022). Targeted genetic inactivation of either SL1 or UBTF was found to prevent the other’s recruitment to the rDNA promoter and to block PIC formation, thus showing that *in vivo* promoter recruitment of these factors was interdependent and likely cooperative. Particularly telling, the loss of SL1 prevented promoter recruitment of UBTF but had no effect on UBTF binding elsewhere across the rDNA. The study further resolved a long-standing question regarding functional differences between the two ubiquitous splice variants UBTF1 and -2. Previous data had shown that UBTF1 was required for rDNA activity (Kuhn et al., 1994; Sanij et al., 2008). We found that *in vivo*, only the longer UBTF1 variant was able to cooperate with SL1 to form the rDNA PIC, despite both UBTF variants binding identically elsewhere across the rDNA NFR (Tremblay et al., 2022). Thus, while both UBTF1 and 2 were implicated in the epigenetic programming of the rDNA, only UBTF1 could support promotion of 47S pre-rRNA synthesis.

## An induced-fit model for RPI PIC formation

**FIGURE 4:**
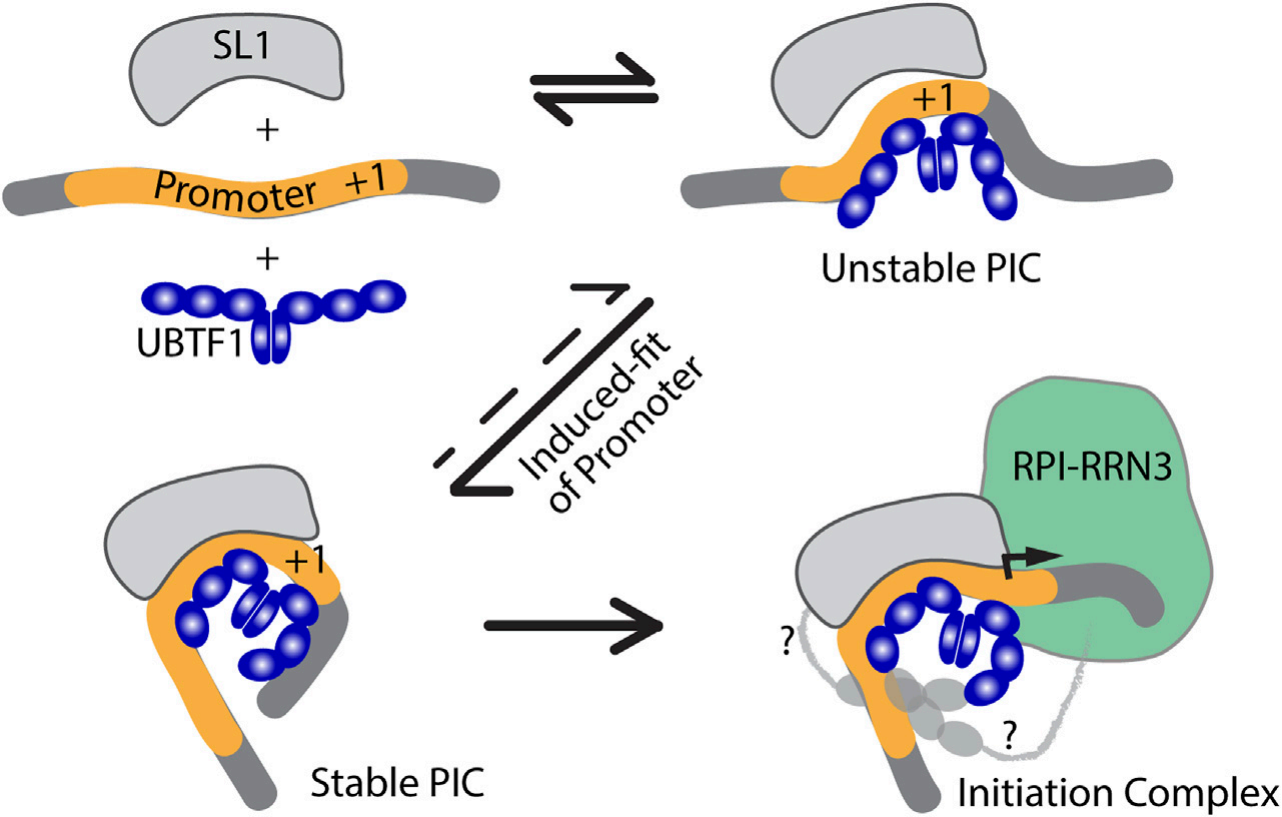
An induced-fit model for RPI PIC formation. Initial interaction of UBTF with the Promoter DNA would transiently induce its bending and provide a topology that improves SL1-DNA interactions, leading to the formation of a stable tripartite SL1-DNA-UBTF PIC structure and subsequent RPI recruitment. In the first three diagrams only the HMGB-boxes 1 to 3 of UBTF required for the Enhancesome fold are shown (blue), while in the last diagram boxes 4 to 6 are indicated along with potential contacts between the C-terminal UBTF acidic tail and RPI (light grey).

The two UBTF variants differ solely within HMGB-box 2, which in UBTF2 lacks a central 37 amino acid sequence (Figures 3A, C). This disrupts the canonical HMGB-box structure and eliminates its ability to recognize bent DNA (Stefanovsky and Moss, 2008). Thus, while UBTF1 could induce a promoter topology resembling the *Enhancesome*, UBTF2 would be unable to induce the DNA bending associated with HMG-box 2 (Figure 3B). This suggested that UBTF-induced promoter topology might provide the “landing” site necessary for SL1 binding and the idea of an “induced-fit” model for PIC formation (Figure 4). In this model, UBTF1 induces a specific topology at the rDNA promoter that SL1 can recognize, bind to and stabilize. In such a model, direct interactions between SL1 and UBTF1 might be of lesser importance than the DNA bending architecture activities of UBTF. In this context, we could detect no protein-protein interaction of SL1 that was selective for UBTF1 over UBTF2 either in cell-free extracts or co-immunoprecipitation of the endogenous factors (Tremblay et al., 2022). It is also worth noting that the rDNA promoter represents a non-preferential site for UBTF binding since, in the absence of SL1, neither UBTF variant was significantly detected at the promoter (Tremblay et al., 2022).

## UBTF variants affect preinitiation complex formation

The independent discovery of two rare UBTF variants in developmental neuroregression obviously pointed to a deregulation of rDNA transcription as the cause. Both the E210K and Q203R variants affected HMGB-box 2 of UBTF further suggested that they might act by affecting PIC formation. A mouse model of the E210K variant indeed displayed a somewhat reduced rate of rDNA transcription. However, most revealing was the finding that this reduction correlated with a reduction in promoter recruitment of both UBTF and SL1 (Tremblay et al., 2022). The E210K and Q203R variants introduced positive charges into helix 1 of HMGB-box2 and it was suggested they might enhance DNA binding by UBTF (Toro et al., 2018; Tinker et al., 2022). However, the observed reduction in PIC formation in the case of E210K rather suggested an effect on promoter recruitment of SL1. Molecular modelling of HMGB-box 2 also showed that the variant K210 side chain would be positioned such that it could not contact DNA and so suggested effects on HMGB-box fold stability or protein-protein interactions with factors such as SL1 rather than interaction with DNA (Tremblay et al., 2022). Unfortunately, similar functional data are not yet available for the Q203R variant but its more severe clinical presentation suggests somewhat stronger effects that may also be mediated by effects on PIC formation.

## UBTF HMGB-box2 instability may be enhanced by the variants

**FIGURE 5:**
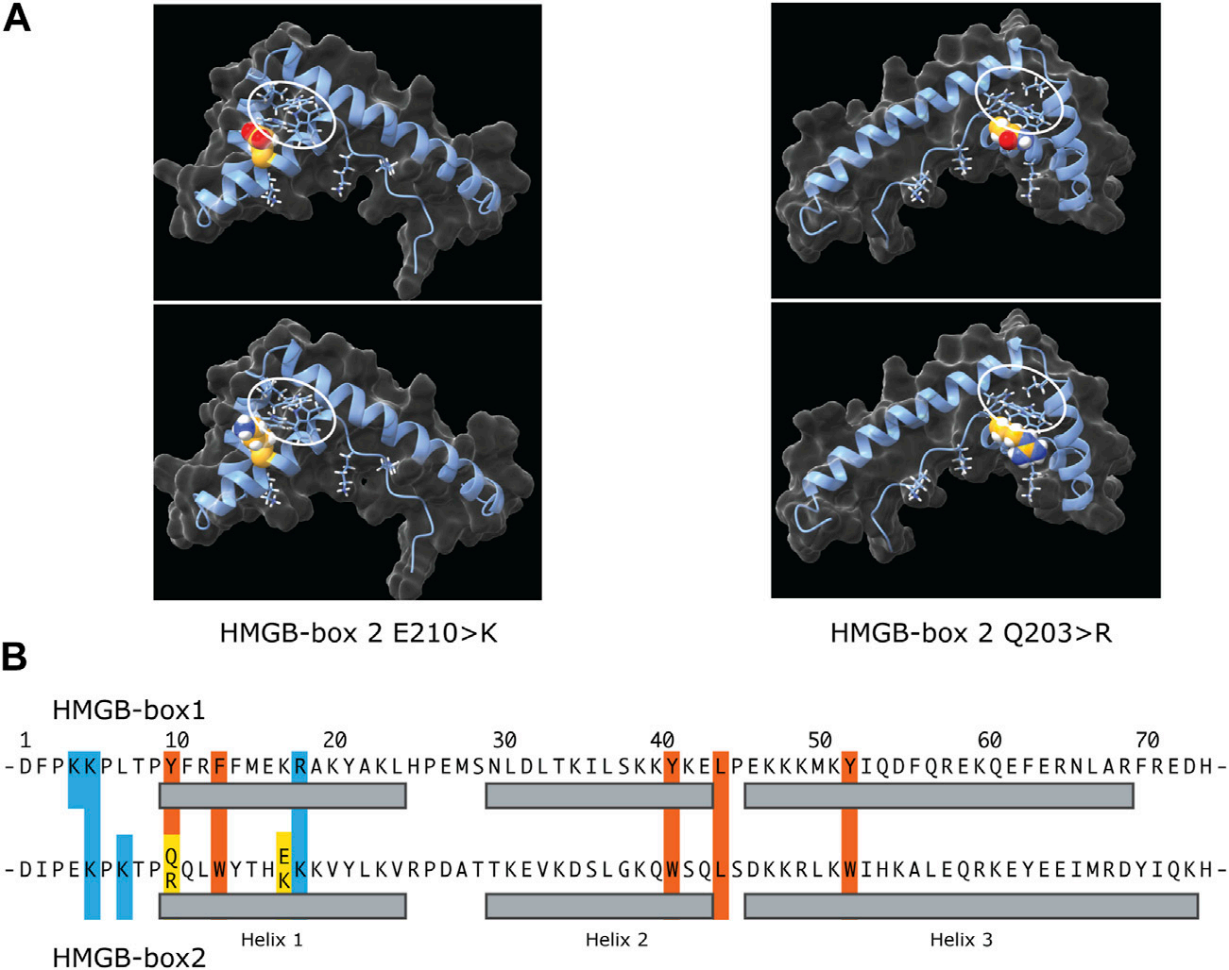
The potential effects of the E210K and Q203R UBTF variants on HMGB-box 2 structure. **(A)** Predicated structures of the variant boxes showing the mutated sidechain (space-filling spheres) and hydrophobic core (ringed). The upper panels show the wild-type box 2 structure predictions and the lower panels the predictions for the variants. In each case the side chains of the affected residues are modelled as space-filling. The two variant structures are shown rotated ∼180 deg. relative to each other to better reveal position of the variant side chains. **(B)** Comparative primary structure alignment of HMGB-box 1 and 2 indicating residues implicated in the respective hydrophobic cores (red) and mutated residues (yellow). Basic residues probably involved in contacting the DNA are also indicated (blue) and their sidechains shown in A.

AlphaFold predictions for the wild type and variant UBTF HMGB-box2 structures do not vary significantly from each other (Matchmaker ChimeraX-1.5 RMSD of 0.3–0.4 Å over 74 alpha-carbons) (Pettersen et al., 2021). However, it should be noted that the AlphaFold algorithm generally underestimates the effects of point variants (Pak et al., 2023). This said, all predicted structures show the typical three helix V-shape fold formed by two near orthogonal wings, a minor wing formed of helix 3 and the antiparallel N-terminus, a major wing composed of helix 1 and 2, and a hydrophobic core formed between these wings that draws mainly from major-wing residues and is key to fold stability (Crane-Robinson et al., 1998) (Figures 5A, B). However, HMGB-box2 of UBTF is unusual in the choice of residues forming its central hydrophobic core that are more usually aromatic in other HMGB-boxes. For example, the HMGB-box 2 sequence of UBTF -tpQQLWy-, residues 8 to 14 in the common HMG-box numbering, corresponds to tpYFRFf in UBTF box 1, -saMFIFs-in UBTF box 4, and to -saFFLFc-of box 2 of the canonical HMGB1 factor. Thus, Q10 of box 2 corresponds to the first of four residues that participate in the hydrophobic core and that are classically mostly aromatic. It is then already unclear whether the wild-type HMGB-box 2 could fold normally or stably in free solution with residues 10 & 11 as QQ. The Q203R variant at position 10 of box 2 at the start of helix 1 would further interfere with the hydrophobic core, making stable folding even less likely. However, when bound to DNA both residues 10 & 11 of box 2, by lying on the surface of the hydrophobic core, might be able to swing out and contact the minor groove bases and hence fulfil a role in DNA binding. This said, variants at the N-terminus of helix 1 of sequence specific HMG-box factors such as SRY are known to significantly affect DNA bending (Murphy et al., 2001). So, the Q203R could have significant implications not only for DNA binding but also DNA bending and like the E210K variant could therefore affect cooperation with SL1. Thus, both the UBTF E210K and Q203R variants likely modify the ability of UBTF1 to correctly adapt the topology of the rDNA promoter to permit stable recruitment of SL1 and efficient preinitiation complex formation.

## So how might UBTF variants lead to neurodegeneration?

It has long been known that cell growth displays a first order dependence on the availability of ribosomes and forms the basis for the so-called growth laws (Scott et al., 2014; Dai and Zhu, 2020). Hence, any variant that negatively affects rRNA synthesis will directly affect growth and broadly impact organism development and homeostasis. Since the UBTF E210K variant reduces preinitiation complex formation and rRNA synthesis, it most probably falls into this category, as we suspect the Q203R variant may also. Why these variants display such distinct physiological effects must for the moment remain a matter of conjecture. However, certain possibilities do suggest themselves.

Specific types of cells and tissues are likely to exhibit enhanced sensitivity to limitations in functional ribosomes, and this may be particularly apparent in the highly specialized post-mitotic cells of the nervous system. Differentiation, cell cycle arrest and aging have all been correlated with reductions in ribosome biogenesis and specifically with reduced rRNA synthesis (Hayashi et al., 2014; Woolnough et al., 2016; Saba et al., 2021). Ribosome assembly is a highly coordinated process beginning co-transcriptionally and defects in the rates of rDNA transcription are known to directly affect its efficiency, leading to imbalances in the 40S and 60S ribosome subunits and the production of inactive ribosomes (Scull and Schneider, 2019). Such defects not only reduce the cellular ribosome complement but also generate inactive ribosomes that interfere with ongoing translation and so have disproportionately severe effects on cell viability.

Partial disruption of ribosome biogenesis also leads to “nucleolar stress”. This is a phenomenon determined in part by the so-called “moonlighting” of excess ribosome components, which activate the p53 and Rb pathways controlling growth (Deisenroth et al., 2016; Lessard et al., 2018; Lessard et al., 2019). But nucleolar stress is also induced by chromosomal rDNA instability linked to defects in transcription. For example, drug arrest of RPI transcription causes severe forms of nucleolar stress that are associated with loss of protective chromatin and the activation of DNA damage responses (Quin et al., 2016; Mars et al., 2020). The nucleosome-loss inherent in rDNA activation leaves these genes susceptible to damage. This “opening” of the rDNA chromatin is in part compensated by UBTF binding but dense loading of RPI transcription complexes also plays an important protective role. Even small reductions in RPI loading can increase rDNA instability, such as was observed when loss of rDNA silencing decreased this loading and enhanced levels of extrachromosomal rDNA, a marker of cellular senescence (Sinclair and Guarente, 1997; Gagnon-Kugler et al., 2009; Kobayashi, 2011). In the case of the E210K fibroblast mouse model, cells responded to the variant by increasing the number of active rDNA copies. While this failed to compensate for the reduced rRNA synthesis, it would have the potentially negative effect of opening more rDNA copies to DNA damage (Tremblay et al., 2022). Thus, defects in rDNA transcription may affect cell viability in complex and very unexpected ways.

Which, if any, of these potential pathways is the fundamental cause of the E210K and Q203R neurodegeneration syndromes is still far from evident. However, the accumulated data to date clearly point to defects in rDNA transcription and suggest it may play a broader role in neurological disease.
